# Modified bar bending method of thoracoscopic nuss procedure on pectus excavatum: a retrospective single-center study

**DOI:** 10.1186/s12887-023-03909-2

**Published:** 2023-03-07

**Authors:** Jichang Han, Yaru Mou, Dongming Wang, Qiongqian Xu, Jian Wang

**Affiliations:** 1 Department of Pediatric Surgery, Qilu Hospital of Shandong University, Jinan, 250012 China; 2 Department of Cardiology, Shandong Provincial Hospital Affiliated to Shandong First Medical University, Jinan, 250021 China

**Keywords:** Pectus Excavatum, Nuss procedure, Funnel chest, Adolescent

## Abstract

**Background:**

Pectus excavatum (PE) is the most common disease of chest wall deformity, with an incidence of 1 in 300—400 births. Nuss procedure has proved to be the best surgical treatment method and has been widely used after clinical use for 30 years. We aimed to review the clinical data of pectus excavatum (PE) of thoracoscopic Nuss procedure adopted the Modified bar bending method of the six-point seven-section type, and compare it with the traditional curved bar bending method to explore the clinical application effect.

**Methods:**

Forty-six cases of clinical data were summarized of children with PE who adopted the treatment of the Modified bar bending method of the six-point seven-section type from January 2019 to December 2021, and 51 cases were compared of PE children who adopted the treatment of traditional curved bar bending method from January 2016 to December 2018, including the data of age, gender, preoperative symptoms, symmetry, Haller index, operation time, bar bending time, intraoperative bleeding, postoperative complications, bar migration, postoperative effect evaluation, etc.

**Results:**

The Procedure duration (*P* = 0.008), bar bending time (*P* < 0.001), and duration of postoperative pain (*P* < 0.001) were reduced significantly, and the incidence of bar migration after surgery was reduced as well by the modified bar bending method. There was no difference compared with traditional Nuss produce, like the incidence of evaluation of postoperative effects (Excellent, *P* = 0.93; Good, *P* = 0.80; Medium, *P* = 1.00; Poor, *P* = 1.00), bar migration (*P* = 1.00), postoperative complications (*P* = 1.00), Clavien- Dindo classification of surgical complications (I = 0.165; II = 1.00; IIIa = 1.00; IIIb = 1.00; VI = 1.00; V = 1.00), operative safety, and operative validity.

**Conclusion:**

Modified bar bending method of the six-point seven-section type, which is a kind of surgical method worth applying and popularizing, and the advantages of minimally procedure duration, bar bending time, and duration of postoperative pain, compared with the traditional bar bending method.

## Background

Pectus excavatum (PE) is a congenital abnormality, which is characterized by the backward bending of the ribs in the middle and lower sternum and the two sides, causing the anterior chest wall to recess backward into a funnel, with the sex ratio tends to be close to 4–6:1 (male: female) and the incidence rate as high as 2–3/1000 [1, 2]. Additionally, familial inheritance has been recorded, with a familial occurrence rate of 54% [3]. Secondary cardiopulmonary dysfunction, chest pain, dyspnoea, and exercise intolerance can be caused by PE [4–7]. Besides, the psychological impact of shame, depression, and distress are well known [7].

Surgical correction remains the definitive management of PE, and many procedures have been applied to correct this malformation. Traditionally, the Ravitch procedure is performed, in which the abnormal cartilage is resected, and the sternum is fractured and fixed in a corrected position [7–9]. In 1998, the "Nuss procedure" was proposed by Donald Nuss, with the feature of minimal trauma and blood loss, shorter operating time with good postoperative results and satisfaction, and a low rate of complications [10, 11]. Nowadays, the Nuss procedure has become the standard operation for pectus excavatum repair, but it also has some disadvantages.

The bar of the traditional Nuss procedure is bent into an arc generally, and the crest of the arc is used to support the lowest point of the sternum for orthopedics [12]. But this bending method has poor support stability and is prone to bar migration and rotation according to our clinical experience. We present a novel modification to the bending method of Nuss procedure which is the modified bar bending method of the six-point seven-section type.

This study aimed to analyze the efficiency of the intraoperative and postoperative outcomes of the novel modification of the Nuss corrective procedure for the treatment of pectus excavatum in young adolescents at our hospital.

Methods

### Patients

The case records of 97 pediatric patients who had undergone traditional or modified method corrective Nuss procedure in the period from January 2016 to December 2021 at the department of pediatric surgery, Qilu Hospital of Shandong University, were retrospectively reviewed. Indications for surgery include severe deformity (Haller Index 3.2 or greater), exercise limitations with exertional dyspnea, chest pain, documented decreased pulmonary function, abnormal exercise tolerance testing, symptomatic cardiac compression, and need for future sternotomy. In addition, significant psychosocial and body-image perception problems would also be taken into account when determining the need for surgery. Inclusion criteria were children of both genders with pectus excavatum that had been treated with the Nuss procedure in our institution during the selected study period. Exclusion criteria were patients who had been operated on using other surgical techniques, intraoperative conversion to any other surgical technique, and patients with incomplete data and who had no possibility of providing a long-term satisfaction grade. This study received approval from the Ethics Review Committee of Qilu Hospital of Shandong University on 31 May 2022 (No. KYLL-202205–013) and was carried out in compliance with the ethical standards established in accordance with the Declaration of Helsinki (2013 edition).

### Outcomes of the study and hypothesis

For the primary objective, we hypothesized the incidence of postoperative bar migration of the modified method performs well, whereas the secondary outcomes of the study were operation time, bar bending time, and duration of postoperative pain.

### Study protocol

The study was designed as a retrospective observational study with a prospective arm where there was active communication with the patients and their parents or legal guardians to determine the grade of satisfaction. To identify the advantage of the modified method over the traditional method, several variables were recorded to investigate which of them could potentially bring an impact on the outcomes (surgery and patient satisfaction). For this purpose, patients treated with the modified bar bending method of the six-point seven-section type were selected as group A and treated by the traditional curved bar bending method were selected as Group B. The following parameters were analyzed for each patient included in study: age, sex, park type, X-Ray, and CT Haller indexes, the total operation time, bar bending time, operation time, intraoperative blood loss, duration of postoperative pain, the postoperative CRP elevation time, postoperative fever time, antibiotic use time, closed pneumothorax, pleural effusion, pneumonia and pleurisy, bar migration, intraoperative and postoperative complications, and evaluation of postoperative effects (1—excellent, 2—good, 3—medium, 4—poor).

### Description of surgery

The patient was under tracheal intubation, general anesthesia, and disinfection routinely who was supine with both upper limbs abduction. The bar size which we selected was measured according to the width of the chest midaxillary lines of the fifth intercostal space.

**Fig. 1:**
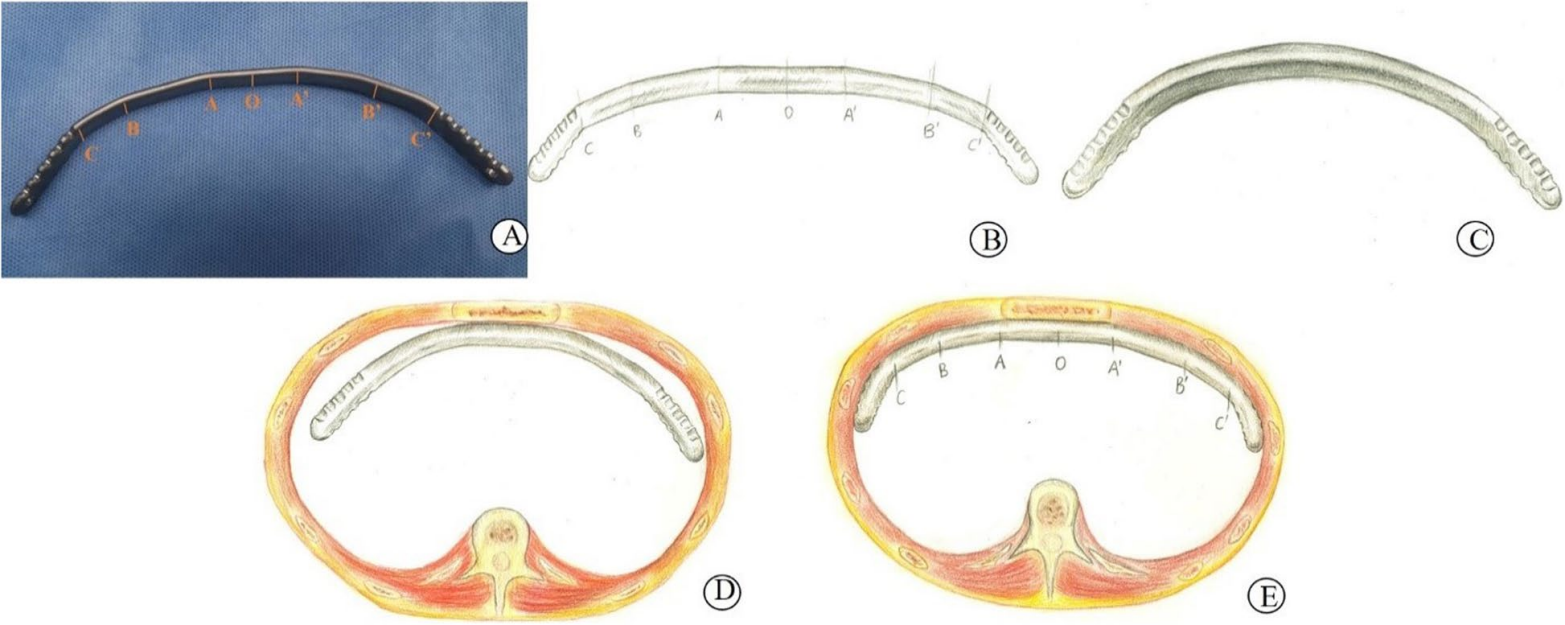
The shape and schematic diagram of the traditional and modified bar bending method. **A** The shape of the modified bar bending method of the six-point seven-section type (O point is the midpoint and A, B, C, A', B', C' are the six bending points). **B** Schematic diagram of the modified bar bending method of the six-point seven-section type. **C** Schematic diagram of the traditional curved bending bar. **D** Schematic diagram of the traditional curved bending bar placed in the sternum. **E** Schematic diagram of the modified bending bar placed in the sternum

The modified bar bending method of the six-point seven-section type is as follows: the median line O of the bar is placed horizontally in the fifth intercostal space in coincidence with the sternal median line. The intersection point of the bar and the fifth intercostal chest wall is the outermost bending point of the bar. The points of the left and right ends are marked as A and A' respectively, and the intercostal area is also the position of the bar entering and leaving the chest cavity. There are 2 bending points between AO and A'O, and the right and left sides are marked as B, C, B', and C' respectively. Bending the bar at the mark of A, B, C, A', B', C' according to the degree of sternal depression and chest wall radian. Note that the outer wings of bars A and A' should fit the corresponding chest wall as closely as possible. After bending, the bar presents a prominent shape of six basis points and seven line segments, with a platform rather than the traditional type of circular arc protruding in the middle. Thus, the method is named six-the point seven-section modified bar bending method (Fig. 1).

**Fig. 2:**
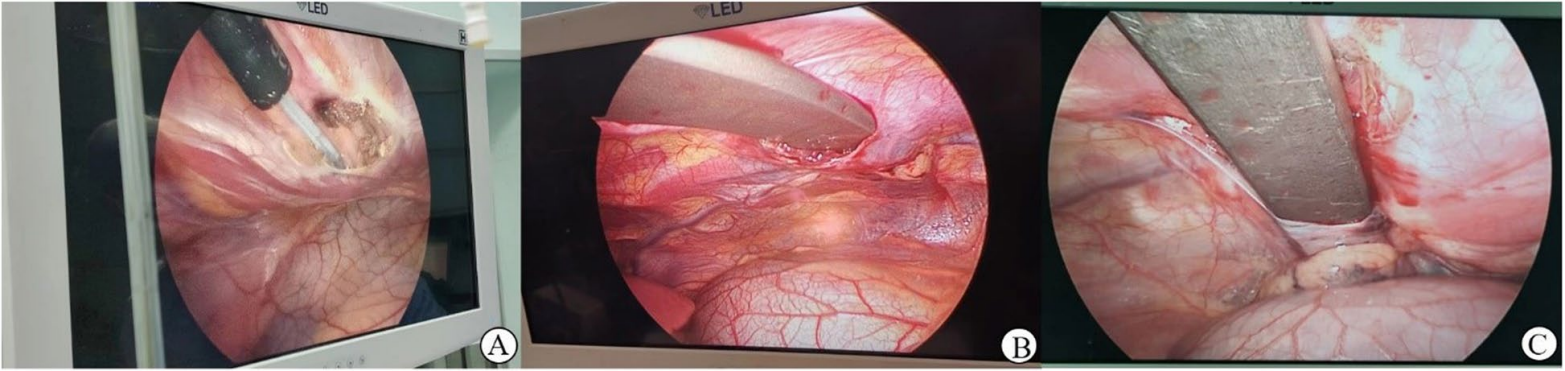
The key step in surgical procedures. **A** Electrocoagulation hook was used to cut open the mediastinum parietal pleural and lib appropriately under thoracoscopic surveillance. **B** The bar is placed through the right thoracic cavity into the mediastinal extrapleural tunnel successfully. **C** Thoracoscopic transfer to the left thoracic cavity to monitor the safe passage of the guide and bar

Nuss procedure was performed with thoracoscopic after the bar was bent. longitudinal incisions were made in the midaxillary line on both sides. Then the muscle fascia was dissociated and the fixed-wing was placed between the muscle and fascia. Next, pneumothorax was established for thoracoscopic access. Under thoracoscopic surveillance, the mediastinal pleura was cut with an electric coagulator hook to allow the guide through the pleura to establish a tunnel and then the guide was penetrated through the left mediastinal pleura to ensure the safety of the pericardium and heart. Note that the position of the guide through the chest cavity should be consistent with the mark point, where the position simulating the bending bar. And then, using a drainage tube to connect the guide and plastically completed orthopedic bar, which was inserted into the sternum through an extraperitoneal tunnel and turned 180 degrees to complete the orthopedic process. After the lung was fully inflated, the two fixed wings were inserted into the outer wings of both bars respectively. Using the No.10 suture fixed to the muscles, which were covered by the muscle fascia, and then closed the incision layer by layer (Fig. 2).

### Follow-up

After the surgical procedure, the patients are observed in the intensive care unit until they are off opioid intravenous medications and after the eventual early complications are resolved. Patient-controlled analgesia (PCA), using morphine or fentanyl, is started in the operating room and is gradually switched to oral pain medications over the next 2–3 days. Other strategies for controlling postoperative pain include oral non-steroidal anti-inflammatory drugs (NSAIDs): ibuprofen, metamizole sodium hydrate, paracetamol, and anxiolytic/spasmolytics such as diazepam. Stool laxatives and emollients are given to prevent constipation while intravenous fluids and proton pump inhibitors are given to reduce the side effects of oral NSAIDs on the gastrointestinal tract [7]. Patients 12 years old and younger are encouraged to wean off all their pain medications by 7 to 10 days from the time of surgery. Teenage patients generally require 2 to 3 weeks. All patients are expected to be off all pain medicine by 4 weeks after the operation [12].

On the first postoperative day, a chest X-Ray is performed to define possible early complications (pneumothorax, subcutaneous emphysema or effusions) and bar position. Pulmonary function is measured during post-operative visits after correction and during visits after bar removal. Follow-up of outpatient visits were performed at 1, 3, 6, 12, 24 months after Nuss surgery and annually until the bar has been removed 2 years. Recurrence is generally evident on physical examination, but is verified radiographically. Patients were screened for surgical complications according to Clavien–Dindo classification [13].

### Statistical methods

Measurement data were tested for normality by Shapiro–Wilk, and data conforming to normal distribution were described by median and interquartile ranges (IQR) and t-test was used. And non-normal distribution was represented by the median (minimum to maximum) and the Mann–Whitney U test was used. The count data were represented by the absolute numbers and percentages, and the comparison between groups was performed by χ2 test and two-sided Fisher exact test, where appropriate. All statistical analyses were performed using SPSS 20.0 software (IBM Corp, Armonk, NY, USA). P < 0.05 was considered statistical significantly.

## Results

Forty-six cases of PE children treated with the modified bar bending method of the six-point seven-section type were selected as group A from January 2019 to December 2021. 29 cases (63%) were divided into type I (symmetric) and 17 cases (37%) were divided into type II (asymmetric) according to Park's classification [14]. 35 cases (76%) were male and 11 cases (24%) were female. The median age was 12 (IQR 5, 13). The median Haller index was 5.63 (IQR 4.08, 7.72). 28 (60.9%) patients were with shortness of breath after exercise.

Fifty-one children with PE treated by the traditional curved bar bending method between January 2016 and December 2018 were selected as Group B, and the case data of this group were summarized above. type I (symmetric) in 34 cases (66.7%), type II (asymmetric) in 17 cases (33.3%), male patients in 40 cases (78.4%), and female in 11 cases (21.6%) among the patients. The median age was 12 (IQR 6, 15). The median Haller index is 5.87 (IQR 4.15, 7.95). 32 (60.9%) patients were with shortness of breath after exercise.

There was no difference in Park type (*P* = 0.71), sex (*P* = 0.78), age (*P* = 0.58), Haller index (*P* = 0.27), or shortness of breath after exercise (*P* = 0.85). The demographic data of the patients are shown in Table 1.

**Table 1:** Demographic data of the patients

Variables	Group A (*n* = 46)	Group B (*n* = 51)	*P* value
	**Modified Method**	**Traditional Method**	
Park type, n (%)			0.71
I type(symmetric)	29 (63%)	34 (66.7%)	
II type(asymmetric)	17 (37%)	17 (33.3%)	
Sex, n (%)			0.78
male	35 (76%)	40 (78.4%)	
female	11 (24%)	11 (21.6%)	
Age, year, median (IQR)	12 (5, 13)	12 (6, 15)	0.58
Haller index, n, median (IQR)	5.63 (4.08, 7.72)	5.87 (4.15, 7.95)	0.27
Shortness of breath after exercise, n (%)	28 (60.9%)	32 (62.7%)	0.85

^*^ Measurement data were used t-test. The count data were calculated by χ2 test

No statistical difference existed among all indicators, no other associated malformation happened, and no serious cardiopulmonary dysfunction or respiratory tract infection symptoms existed before surgery. Surgery for groups was performed by the same treatment group and a bar was inserted above the two groups. Both groups were operated on without massive bleeding successfully. The intraoperative, postoperative, and follow-up data were summarized and analyzed as follows.

The median (IQR) procedure duration was significantly decreased in group A than Group B( 90 (IQR 70, 110) VS 90 (IQR 70, 110), *p* = 0.008), which is due to the decrease in bar bending time (14 (IQR 10, 16) VS 23 (IQR 20, 27), *P* < 0.001). Besides, there was no difference in operation time (74 (IQR 65, 82) VS 75 (IQR 70,85), *P* = 0.13) and intraoperative blood loss (6 (IQR 3, 8) VS 6 (IQR 4, 8), *P* = 0.23) between the two groups. The Comparison of intraoperative data is shown in Table 2.

**Table 2:** Comparison of intraoperative data

Variables	Group A (*n* = 46)	Group B (*n* = 51)	*P* value
	**Modified Method**	**Traditional Method**	
Procedure duration, min, median (IQR)	90 (65, 100)	100 (70, 110)	0.008
Bar bending time, min, median (IQR)	14 (10, 16)	23(20, 27)	P < 0.001
Operation time, min, median (IQR)	74 (65, 82)	75 (70,85)	0.13
Intraoperative blood loss, ml, median (IQR)	6 (3, 8)	6 (4, 8)	0.23

^*^ Measurement data were used t-test. The count data were calculated by χ2 test

The postoperative CRP elevation time (4 (IQR 3,6) VS 5 (IQR 4,6), *P* = 0.72), the duration of postoperative fever time (3.1 ± 1.1 VS 3.6 ± 1.5, *P* > 0.05), and the duration of antibiotic use (7.3 ± 2.1 VS7.4 ± 2.8, *P* > 0.05) were similar between the two groups without statistical difference. However, the duration of postoperative pain in group A was shorter than that in group B (3.1 ± 1.2 VS 7.5 ± 1.3, *P* < 0.05). The list of Outcomes after treatment is shown in Table 3.

**Table 3:** Outcomes after treatment

Variables	Group A (*n* = 46)	Group B (*n* = 51)	*P* value
	**Modified Method**	**Traditional Method**	
duration of postoperative pain, day	3 (2,4)	7 (6,8)	*P* < 0.001
The postoperative CRP elevation time, day	4 (3,6)	5 (4,6)	0.72
Postoperative fever time, day	3 (2,4)	4 (3,5)	0.07
Antibiotic use time, day	7 (6,9)	7 (5,10)	0.84
Evaluation of postoperative effects			
Excellent	33/46 (71.1%)	37/51 (72.5%)	0.93
Good	10/46 (21.7%)	10/51 (19.6%)	0.80
Medium	3/46 (6.5%)	4/51 (7.8%)	1.00
Poor	0	0	1.00

^*^ Measurement data were used t-test. The count data were calculated by χ2 test and two-sided Fisher exact test, where appropriate

The evaluation criteria for postoperative effects are as follows:

- (1) Comparison of chest X-ray lateral radiographs before and after the operation.
- (2) The flatness and symmetry of thoracic cavity appearance.
- (3) Satisfaction degree of children and their families.
- (4) Thorax fullness, extension, and elasticity.

**Fig. 3:**
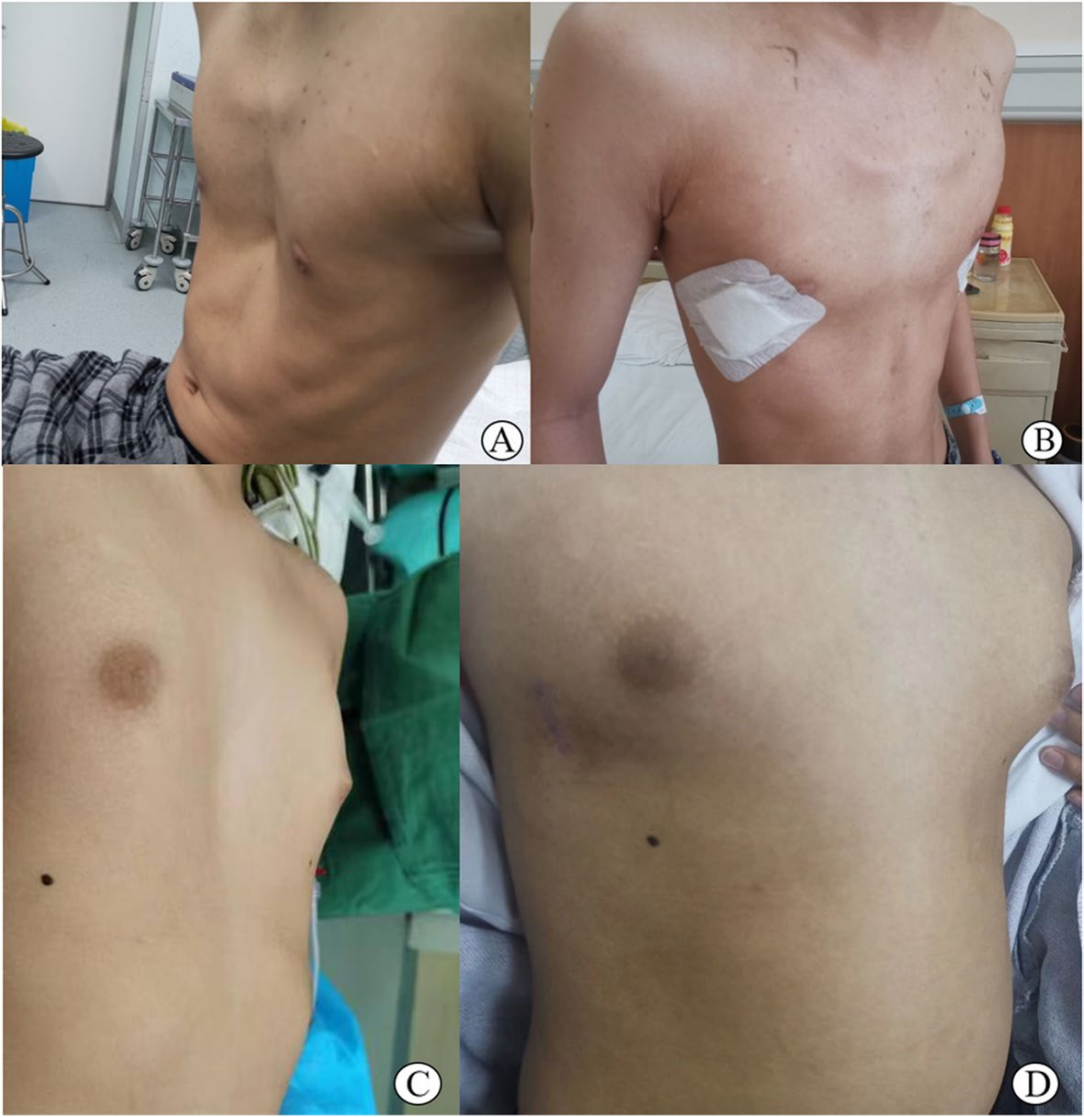
**A** Preoperative presentation of a male patient with pectus excavatum scheduled for corrective Nuss procedure. **B** Postoperative result of the same patient after surgery. **C** Preoperative presentation of a female patient with pectus excavatum scheduled for corrective Nuss procedure. **D** Postoperative result of the same patient after surgery

**Fig. 4:**
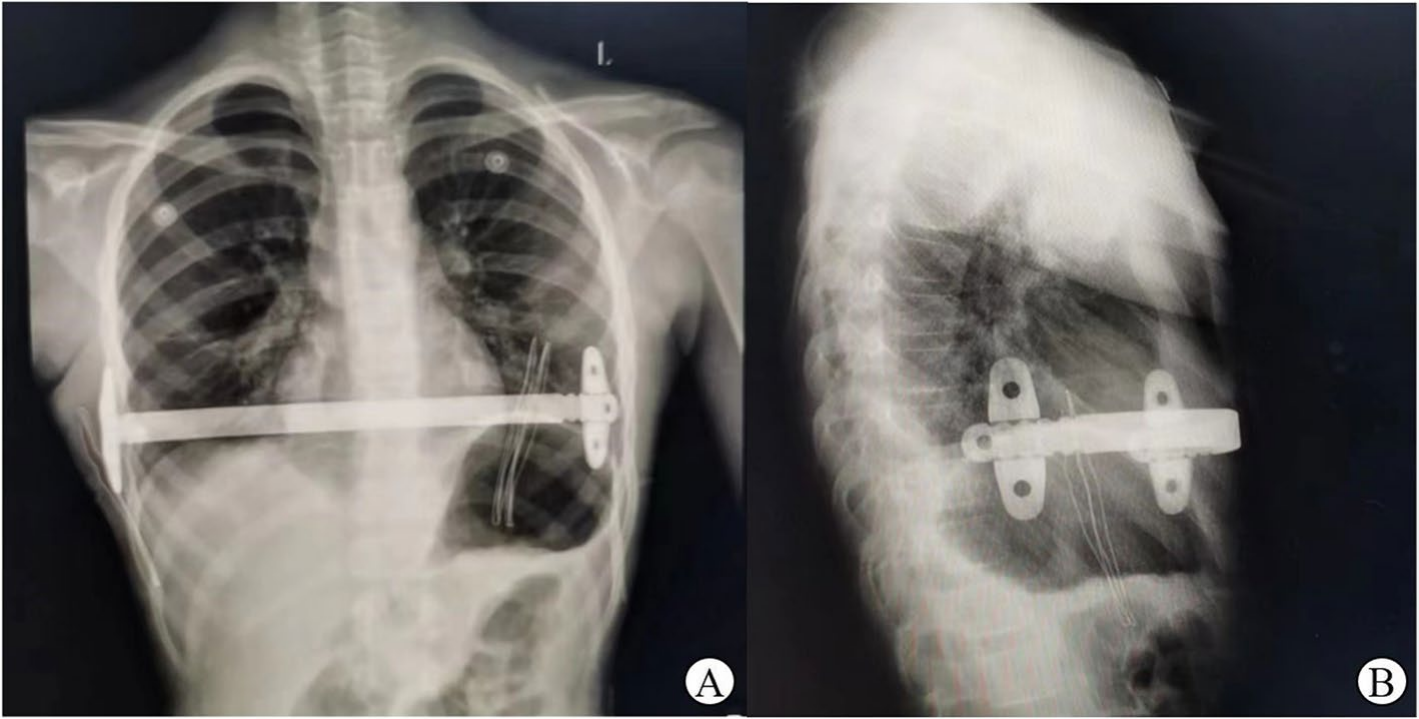
Bar position at postoperative chest X-ray: posteroanterior (**A**) and laterolateral (**B**) views

If it meets 4 criteria, it is preferable; 3 are good; 2 are medium; 0 to 1 is poor. As a result, there was no "poor" case in the A and B groups, and there was no statistical difference in the proportion of "excellent", "good" and "medium" cases according to the above evaluation criteria (Fig. 3) (Fig. 4).

Early complications were recorded in 19 patients (19.6%), and late complications were reported in 4 (4.1%), while intraoperative complications were not recorded. The most common early complications were closed pneumothorax, followed by pneumonia and pleurisy and poor healing of incision, while two patients had pleural effusion. The most common late complication was bar migration.

For common complications, there was no difference in closed pneumothorax (3 (6.5%) VS 4 (7.8%), *P* = 1.00), pleural effusion (1 (2.2%) VS 1 (2.0%), *P* = 1.00), pneumonia and pleurisy (2 (4.3%),4.3% VS 3 (5.9%), P = 1.00) and the incidence of poor incision healing (2 (4.3%) VS 3 (5.9%), *P* = 1.00) between two groups. As for bar migration, no case happened in group A and 4 (7.8%) cases happened in group B, and there was no difference between the two groups (*P* = 1.00). The list of complications by patient is shown in Table 4.

**Table 4:** Early and late postoperative complications after Nuss procedure

	**Group A (*n* = 46), n (%)**	**Group B (*n* = 51), n (%)**	**Total, n (%)**	***P* value**
	**Modified Method**	**Traditional Method**		
Early Complications				
Closed pneumothorax	3 (6.5%)	4 (7.8%)	7 (7.2)	1.00
Pleural effusion	1 (2.2%)	1 (2.0%)	2 (2.1)	1.00
Pneumonia and pleurisy	2 (4.3%)	3 (5.9%)	5 (5.2)	1.00
Poor healing of incision	2 (4.3%)	3 (5.9%)	5 (5.2)	1.00
Late Complications				
Bar migration	0 (0)	4 (7.8%)	4 (4.1)	1.00

^*^The data were calculated two-sided Fisher exact test

The patients with closed pneumothorax and pleural effusions were treated conservatively (antibiotic and corticosteroid therapy) without the need for thoracal drainage and resolved within two weeks. Five cases of pneumonia and pleurisy were treated conservatively with antibiotics. Additionally, all four late bar migrations were less than 40 degrees of rotatory migration (rotational bar movement-torsion) and did not need surgical reintervention. Surgical complications according to Clavien–Dindo classification are shown in Table 5.

**Table 5:** Clavien- Dindo classification of surgical complications

Grade	Group A (*n* = 46), n (%)	Group B (*n* = 51), n (%)	*P* value	Total
	**Modified Method**	**Traditional Method**		
I	8 (17.4)	15 (29.4)	0.165	23(23.7)
II	0 (0)	0 (0)	1.00	0 (0)
IIIa	0 (0)	0 (0)	1.00	0 (0)
IIIb	0 (0)	0 (0)	1.00	0 (0)
VI	0 (0)	0 (0)	1.00	0 (0)
V	0 (0)	0 (0)	1.00	0 (0)

^*^ The data were calculated by χ2 test and two-sided Fisher exact test, where appropriate

## Discussion

Pectus excavatum (PE) is the most common disease of chest wall deformity. Although the Nuss procedure has been widely carried out around the world some improvements based on the traditional Nuss procedure can still bring benefits to patients [15]. This study proved that compared with the traditional Nuss procedure, the modified bar bending method of the thoracoscopic Nuss procedure was reduced significantly in the procedure duration, bar bending time, and duration of postoperative time. And there was no difference in the evaluation of postoperative effects, bar migration, postoperative complications, Clavien- Dindo classification of surgical complications, operative safety, and operative validity, compared with traditional Nuss procedure.

Since the introduction of the Nuss procedure, many modifications have been proposed to improve outcomes and safety. But the improvement regarding the bending step was adequate. In our study, several cases of postoperative complications of bar migration happened in traditional Nuss procedure, which prompted us to analyze the phenomenon. The traditional bar is bent into an arc shape, which overemphasizes sternum elevation in the central position [12]. And owing to the power of the sternum elevation being concentrated, the duration of postoperative pain is longer. Besides, the circular shape is required to bend the whole bar severally with bending equipment to adapt to the chest shape, which takes a long time and does more harm to the patients. For children before adolescence, bar shifting incidence is lower on account of the hardness of the sternum and ribs is lower slightly. And the probability of bar migration increased significantly for adolescents alone with the bone growth solidified and muscles getting strong [16]. Thus, the design of the bar could sustain the hard sternum is important affairs. Given the above defects, we improved the design of the modified bar bending method, which is the six-point seven-section type. The advantages of this modified method are as follows:

- (1) The design of the prominent high point in the center of the bar is changed into the platform shape, which is fully fitted with the dorsal sternum plane, improve stability, and reduce postoperative pain.
- (2) The whole bar will no longer be folded into an arc shape gradually, and only the six fixed points will be bent. Thus, the bending time of the bar decreased dramatically.

Consulting literature, a series of modifications focused on the parts of measuring the chest, bar selection and configuration, marking the patient, thoracoscopy, subcutaneous tunneling, substernal tunneling, sternal elevation, bar insertion and rotation, bar fixation, pneumothorax evacuation [17]. Anthony I. Squillaro et al. [18] emphasized the importance of chest introducer entry and exit sites as a critical operative step, which is a novel alternative approach to asymmetric pectus excavatum repair. Guoqing Li [19, 20] introduced the modified Nuss procedure operating with right unilateral thoracoscopy and a new bar of fixed bend, which overcomes the necessity of bar turning over with the advantages of less invasive and more safety. The median of total operating time in this article is 70 min, which is shorter than our modified procedure. And the difference also confirm that the time of bending bar cost much time. A. Durry, W. Rainey Johnson, R. Kabbaj et al. [21–23] added a subxiphoid incision to improve the visualization of the anterior mediastinum and limit the risk of cardiac or pericardial perforation. But the duration of operation gets longer which could generate complications. There is no difference between the Thoracoscopic extrapleural modification of the Nuss procedure and the standard Nuss procedure [24]. KristaLai et al. [25] described cryoablation as an effective method of postoperative analgesia, and the expectation is that patients will be off opioid analgesics by 1-week postoperatively. And applying cryoablation could decrease the length of stay, control pain, and result in overall cost savings.

While this study proposes a novel alternative bending approach to pectus excavatum repair, the nature of a technical report from a single center and single surgeon experience are key limitations to this technical report. Several items like the incidence of bar migration in our patients and how they relate to the success of our modified method have yet to be analytically examined. A multiple-center prospective study with a large number of cases will be needed to further draw our conclusion.

## Conclusion

In conclusion, the modified bar bending method confirms fixed points to bend the bar, which reduces the operation time and duration of postoperative pain, while the safety, the incidence of postoperative bar migration, effectiveness, and satisfactory effect remain the same. Apart from this, the modified method shows the characteristics of a minimally invasive Nuss procedure. Thus, the modified surgical method is worthy of popularization and application.

## Data Availability

The datasets used and analyzed during the current study available from the corresponding author on reasonable request.
